# Developing trustworthy artificial intelligence: insights from research on interpersonal, human-automation, and human-AI trust

**DOI:** 10.3389/fpsyg.2024.1382693

**Published:** 2024-04-17

**Authors:** Yugang Li, Baizhou Wu, Yuqi Huang, Shenghua Luan

**Affiliations:** ^1^CAS Key Laboratory for Behavioral Science, Institute of Psychology, Chinese Academy of Sciences, Beijing, China; ^2^Department of Psychology, University of the Chinese Academy of Sciences, Beijing, China

**Keywords:** trustworthy AI, interpersonal trust, human-automation trust, human-AI trust, AI ethics, warmth, competence, trust measurement

## Abstract

The rapid advancement of artificial intelligence (AI) has impacted society in many aspects. Alongside this progress, concerns such as privacy violation, discriminatory bias, and safety risks have also surfaced, highlighting the need for the development of ethical, responsible, and socially beneficial AI. In response, the concept of trustworthy AI has gained prominence, and several guidelines for developing trustworthy AI have been proposed. Against this background, we demonstrate the significance of psychological research in identifying factors that contribute to the formation of trust in AI. Specifically, we review research findings on interpersonal, human-automation, and human-AI trust from the perspective of a three-dimension framework (i.e., the trustor, the trustee, and their interactive context). The framework synthesizes common factors related to trust formation and maintenance across different trust types. These factors point out the foundational requirements for building trustworthy AI and provide pivotal guidance for its development that also involves communication, education, and training for users. We conclude by discussing how the insights in trust research can help enhance AI’s trustworthiness and foster its adoption and application.

## 1 Introduction

Artificial intelligence (AI) is the driving force behind industry 4.0 and has profoundly affected manufacturing, business, work, and our daily life (Magd et al., 2022). The invention of generative AI technologies, such as ChatGPT, marks a particularly significant leap in AI’s competence. While bringing significant changes to society, the development of AI has also sparked various concerns, including privacy invasion, hidden biases and discrimination, security risks, and ethical issues (Yang and Wibowo, 2022). One response to these concerns is the emergence of and emphasis on *trustworthy AI* that aims to strike a good balance between technological advancement and societal and ethical considerations (Li et al., 2023).

Trustworthy AI, defined as AI that is lawful, ethical, and robust [High-Level Expert Group on Artificial Intelligence (AI HLEG), 2019], represents a critical focus on responsible technology deployment. To develop trustworthy AI, multiple countries and international organizations have issued guidelines. For instance, the European Union issued the “Ethics Guidelines for Trustworthy AI” in April 2019 (High-Level Expert Group on Artificial Intelligence (AI HLEG), 2019); China published the “Governance Principles for a New Generation of Artificial Intelligence: Develop Responsible Artificial Intelligence” in June 2019 (National Governance Committee for the New Generation Artificial Intelligence, 2019); and on October 30, 2023, President Biden of the United States signed an executive order on the “safe, secure, and trustworthy development and use of artificial intelligence” (The White House, 2023). These guidelines lay out requirements for the development of AI that ensure safety and protect privacy, enhance transparency and accountability, and avoid discrimination.

Trust and trustworthiness are key psychological constructs that have been extensively explored in research on interpersonal, human-automation, and human-AI trust, providing many insights on how a person or an agent can become trustworthy. The aforementioned guidelines primarily outline requirements for developers and providers of AI, but do not pay sufficient attention to how end-users may develop trust in AI systems. Research on trust specifies users’ expectations of AI, thus aiding the comprehension of their concerns and needs. It also assists in identifying which attributes of AI systems are crucial for establishing trust and improving their design. Furthermore, because trust has a significant impact on the adoption of AI, trust research may also help enhance the public’s acceptance and adoption of AI technology.

In this paper, we apply trust theories to the context of trustworthy AI, aiming to shed lights on how to create reliable and trustworthy AI systems. In doing so, this paper makes several notable contributions to the field of AI trust research. First, we systematically review research on interpersonal, human-automation, and human-AI trust by viewing the perception of AI from the perspective of social cognition (Frischknecht, 2021). It serves to validate and build upon theoretical frameworks in previous literature reviews and meta-analyses from a broader, more coherent, and more inclusive angle. Second, based on a three-dimension framework of trust that encompasses trustor, trustee, and their interactive context, we compile and summarize a large number of factors that may influence trust in AI by reviewing a wide range of empirical studies. Third, by identifying and consolidating these influencing factors, our paper offers guidance on enhancing AI trustworthiness in its applications, bridging theoretical concepts and propositions with practical applications.

Overall, we aim to build a comprehensive framework for understanding and developing trustworthy AI that is grounded in end-users’ perspectives. Here, we focus primarily on the formation of trust, and do not distinguish between specific applications in automation or AI but refer to them collectively as automation or AI (Yang and Wibowo, 2022). The following sections are organized according to the three types of trust, ending with a discussion on the implications of trust research on enhancing trustworthy AI.

## 2 Interpersonal trust

Rotter (1967) first defined interpersonal trust as the trustor’s generalized expectancy for the reliability of another person’s words or promises, whether verbal or written. This generalized expectancy, commonly known as trust propensity, is considered a personality characteristic that significantly influences actual behavior (Evans and Revelle, 2008). Trust typically arises in contexts characterized by risk and uncertainty. Mayer et al. (1995) framed interpersonal trust as a dyadic relationship between a trustor, the individual who extends trust, and a trustee, the entity being trusted, and treated trust as the willingness of the trustors to make themselves vulnerable despite knowing that the trustees’ actions could significantly impact them and irrespective of the trustors’ ability to monitor or control those actions. This section outlines critical factors that shape interpersonal trust.

### 2.1 Characteristics of interpersonal trust

Trust is a term frequently encountered in daily life, characterized by a multitude of definitions and generally regarded as a multidimensional concept. McAllister (1995) identified two dimensions of interpersonal trust: cognitive and affective, while Dirks and Ferrin (2002) expanded this to include vulnerability and overall trust. Jones and Shah (2016) further categorized trust into three dimensions: trusting actions, trusting intentions, and trusting beliefs. Different theories capture distinct characteristics of trust but exhibit several key commonalities.

First, trust is dyadic, involving a trustor and a trustee, each with certain characteristics. From a dyadic perspective, interpersonal trust can be categorized into three types: reciprocal trust, highlighting the dynamic interactions between parties; mutual trust, reflecting a shared and consistent level of trust; and asymmetric trust, indicating imbalances in trust levels within interpersonal relationships (Korsgaard et al., 2015). The propensity to trust of the trustors exhibits significant individual differences, influenced by gender (Dittrich, 2015; Thielmann et al., 2020), age (Bailey et al., 2015; Bailey and Leon, 2019), and personality traits (Ito, 2022). Trust propensity influences trust at an early stage, and the assessment of the trustworthiness of trustees (trust beliefs) may ultimately determine the level of trust (McKnight et al., 1998).

Second, interpersonal trust can be influenced by interactive contexts, such as social networks and culture (Baer et al., 2018; Westjohn et al., 2022). The trustworthiness of strangers is frequently evaluated through institutional cues, including their profession, cultural background, and reputation (Dietz, 2011). Third, trust occurs within uncertain and risky contexts, and it is closely linked to risk-taking behavior (Mayer et al., 1995). Fourth, trust is usually dynamic. Trust propensity embodies a belief in reciprocity or an initial trust, ultimately triggering a behavioral primitive (Berg et al., 1995). Trustors will determine whether to reinforce, decrease, or restore trust based on the outcomes of their interactions with trustees. Individuals form expectations or anticipations about the future behavior of the trusted entity based on various factors, such as past experience and social influences. Thus, the trust dynamic is a process of social learning that often evolves gradually and changes with interactive experiences (Mayer et al., 1995).

The above analysis shows that factors influencing interpersonal trust can be examined from three dimensions: the trustor, the trustee, and their interactive context. Interpersonal interactions correlate with changes in variables related to these three dimensions, ultimately leading to variations in the levels of trust and actual behavior.

### 2.2 Measurement of interpersonal trust

Trust, especially trust propensity, can be quantified using psychometric scales. These scales evaluate an individual’s specific trust or disposition toward trusting others via a set of questions (Frazier et al., 2013). Example items include, “I am willing to let my partner make decisions for me” (Rempel et al., 1985), and “I usually trust people until they give me a reason not to trust them” (McKnight et al., 2002). Meanwhile, economic games, such as trust game, dictator game, public goods game, and social dilemmas, provide a direct and potentially more accurate means to assess trust by observing individual actions in well-defined contexts (Thielmann et al., 2020). This approach is beneficial for deducing levels of trust from real decisions and minimizing the impact of social desirability bias (Chen et al., 2023). When integrated, behavioral observations from economic games and self-reported beliefs yield a more comprehensive perspective on trust by combining the strengths of observed actions and declared beliefs.

The aforementioned methods represent traditional approaches to assessing interpersonal trust. Alternative methods for measuring interpersonal trust also exist. To avoid redundancy, these methods are reviewed in the “Measurement of Trust in Automation” section.

## 3 Trust in automation

Trust extends beyond human interactions. Its importance is notable in interactions between humans and automated systems (Hoff and Bashir, 2015). According to Gefen (2000), trust in automation is the confidence, based on past interactions, in expecting actions from automation that align with one’s expectations and benefit oneself. In a similar vein, Lee and See (2004) characterize trust in automation as a quantifiable attitude that determines the extent of reliance on automated agents. Consequently, human-automation trust, akin to interpersonal trust, constitutes a psychological state that influences behaviors.

Trust is crucial for the adoption of automation technologies, and a deficiency of trust in automation can lead to reduced reliance on these systems (Lee and See, 2004). Since the 1980s, with the widespread adoption of automation technology and its increasing complexity, research in human-automation interaction, technology acceptance models, and human-automation trust has drastically expanded. This section offers a brief overview of research in this field.

### 3.1 Automation

Automation usually refers to the technology of using devices, such as computers, to replace human execution of tasks in modern society, where automated technologies increasingly take over functions for efficiency, accuracy, and safety purposes (Kohn et al., 2021). Based on system complexity, autonomy, and necessary human intervention, automation can be divided into 10 levels, with level 0 signifying full manual control and level 10 denoting complete automation (Parasuraman et al., 2000). Furthermore, Parasuraman et al. (2000) identified four principal functions of automation within a framework of human information processing: information acquisition, information analysis, decision making, and action execution. An automation system may exhibit varying degrees of automation across these distinct functions.

The field of human-automation interaction has evolved alongside advances in computer technology, as reflected in the progress of its terminology: from HCI (Human-Computer Interaction) and HRI (Human-Robot Interaction) to HAI (Human-AI Interaction) (Ueno et al., 2022). Initially, research on automation trust was concentrated in sectors such as military, aviation, banking, and industrial manufacturing. With advancing computer technology, the focus of automation trust research has expanded to encompass office settings and the service sector. Furthermore, in the context of the internet, the pivotal importance of trust in the adoption of e-commerce, e-governance, and social media platforms has also been extensively investigated (Gefen, 2000; Featherman and Pavlou, 2003; Khan et al., 2014).

### 3.2 Similarities between interpersonal trust and human-automation trust

Being a cornerstone of sustained human cooperation, trust is equally crucial to human-automation collaboration (Xie et al., 2019). Trust in humans and automation shares similarities, supported by both empirical and neurological evidence (Lewandowsky et al., 2000; Hoff and Bashir, 2015). For instance, a three-phase experiment study by Jian et al. (2000) that included tasks of word elicitation and meaning comparison showed that the constructs of trust in human-human and human-automation interactions are analogous. This resemblance may stem from the similar perceptions that individuals hold toward automated agents and fellow humans (Frischknecht, 2021).

Despite their non-human appearance, computers are often subject to social norms and expectations. Nass et al. (1997) demonstrated that assigning male or female voices to computers elicits stereotypical perceptions. In a similar vein, Tay et al. (2014) reported that robots performing tasks aligned with gender or personality stereotypes—such as medical robots perceived as female or extroverted, and security robots as male or introverted—received higher approval ratings. Moreover, studies in economic games like the ultimatum and public goods games have shown that people display prosocial behaviors toward computers, suggesting a level of social engagement (Nielsen et al., 2021; Russo et al., 2021).

The Computers Are Social Actors (CASA) paradigm posits that during human-computer interactions, individuals often treat computers and automated agents as social beings by applying social norms and stereotypes to them (Nass et al., 1997). Such anthropomorphization usually happens subconsciously, leading to automated agents being perceived with human-like qualities (Kim and Sundar, 2012). In reality, intelligent devices exhibit anthropomorphism by mimicking human features or voices, setting them apart from traditional automation (Troshani et al., 2021; Liu and Tao, 2022). Although AI lacks emotions and cannot be held accountable for its actions, it is usually perceived through the lens of social cognition, making it difficult to regard AI as merely a machine or software; instead, AI is often viewed as an entity worthy of trust (Ryan, 2020).

### 3.3 Importance of trust in automation

Automation differs significantly from machines that operate specific functions entirely and indefinitely without human intervention (Parasuraman and Riley, 1997). For example, traditional vehicle components such as engines, brakes, and steering systems are generally regarded as highly reliable; in contrast, autonomous vehicles often evoke skepticism regarding their capabilities (Kaplan et al., 2021). While tasks performed by automation could also be executed by humans, the decision to rely on automation is contingent upon trust. For instance, individuals may refrain from using a car’s autonomous driving feature if they distrust its reliability. Moreover, the complexity of automation technologies may lead to a lack of full understanding by users (Muir, 1987), a gap that trust can help to bridge. Additionally, automation systems are also known to be particularly vulnerable to unexpected bugs (Sheridan, 1988), making the effectiveness of such systems heavily reliant on users’ trust in their performance (Jian et al., 2000).

Because of different individual understandings of automation and the complexity of automated systems, people may exhibit inappropriate trust in automation (Lee and See, 2004), which may lead to misuse or disuse. Misuse refers to the inappropriate use of automation, such as automation bias, that is, people relying on the recommendations of automated systems too much instead of exercising careful information search and processing. They may ignore information that contradicts the suggestions of the automation system, even if that information may be correct (Parasuraman and Manzey, 2010). Disuse refers to people refusing to use automation (Venkatesh et al., 2012). For instance, in decision-making tasks, algorithm aversion, which refers to skepticism toward algorithms, the core of automated systems, often takes place (Burton et al., 2020). The lack of public acceptance impedes advanced technology from achieving its full potential and practical application (Venkatesh et al., 2012). Similarly, inappropriate trust compromises the effectiveness of automated systems. Aligning the public’s trust level with the developmental stage of automation represents an ideal scenario. Thus, it is crucial to investigate the factors that shape trust in automation.

In human-automation trust, viewing trust as an attitude is widely accepted. However, in the context of interpersonal trust, the term *attitude* is not often used, whereas *willingness* is commonly employed. This distinction likely stems from technology acceptance theories, which posit that attitude shapes behavioral intentions and in turn influences actual behavior (Gefen et al., 2003). Hence, the importance of trust in automation is underscored by its effect on users’ behavior toward automated systems.

### 3.4 Measurement of trust in automation

Kohn et al. (2021) conducted a comprehensive review of methods for measuring human trust in automation, classifying these into self-reports, behavioral measures, and physiological indicators. Self-report methods typically involve questionnaires and scales, while behavioral measures include indicators like team performance, compliance and agreement rate, decision time, and delegation. Despite varied terminologies, these measures are based on the same principle: individuals demonstrating trust in an automation system are more inclined to follow its recommendations, depend on it, comply with its advice, lessen their oversight of the system, and delegate decision-making authority to it. Such behaviors are more evident when the automation system demonstrates high accuracy, potentially improving group performance. In dual-task situations, systems that are trusted usually result in faster decision-making and response times for ancillary tasks, whereas distrust can lead to slower responses.

Physiological indicators include those from skin conductance, EEG (electroencephalography), fMRI (functional magnetic resonance imaging), and fNIRS (functional near-infrared spectroscopy). A notable finding is that a reduction in skin conductance, suggesting lower cognitive load and emotional arousal, is associated with increased trust in automation (Khawaji et al., 2015). Moreover, employing methodologies like EEG, fMRI, and fNIRS to investigate the brain regions engaged in processing trust has demonstrated notable alterations (Ajenaghughrure et al., 2020).

Self-reporting methods, such as questionnaires measuring trust, capture static aspects of trust but cannot reflect its dynamic nature. Ayoub et al. (2023) introduced a dynamic measurement of trust during autonomous driving. Drivers’ physiological measures, including galvanic skin response, heart rate indices, and eye-tracking metrics, are recorded in real-time. Machine learning algorithms were then used to estimate trust based on these data. This real-time assessment of trust is critical for capturing its dynamic changes, thereby facilitating trust calibration.

### 3.5 The relationship between automation and AI

#### 3.5.1 AI: a next generation of automation

AI typically refers to the simulation of human intelligence by computers (Gillath et al., 2021). This simulation process encompasses learning (acquiring information and using it to acquire rules), reasoning (using rules to reach conclusions), and self-correction. In essence, AI represents a sophisticated form of automation, enhancing its domain and efficacy. In this paper, we refer automation as traditional automated technologies that are distinct from AI. The distinction lies in automation being systems that perform repetitive tasks based on static rules or human commands, while AI involves systems skilled in dealing with uncertainties and making decisions in novel situations (Cugurullo, 2020; Kaplan et al., 2021).

Interestingly, in the initial research on human-automation interaction, AI was considered a technology difficult to implement (Parasuraman and Riley, 1997). However, in the 21st century and especially after 2010, AI technology has progressed significantly. Nowadays, the impact of AI technology and its applications pervade daily life and professional environments, encompassing speech and image recognition, autonomous driving, smart homes, among others. AI can autonomously deliver personalized services by analyzing historical human data (Lu et al., 2019). Particularly, the recent advancements in generative AI have provided a glimpse of the potential for achieving general AI. That said, understanding how current AI arrives at specific decisions or outcomes can be complex due to its reliance on vast amounts of data and intricate algorithms.

#### 3.5.2 From trust in automation to trust in AI

With the advancement and widespread applications of AI, trust in AI has indeed become a new focal point in the study of human-automation interaction. This transition redefines relationships between humans and automation, moving from reliance on technologies for repetitive, accuracy-driven tasks to expecting AI to demonstrate capabilities in learning, adapting, and collaborating (Kaplan et al., 2021). This evolution in trust requires AI systems to demonstrate not only technical proficiency but also adherence to ethical standards, legal compliance, and socially responsible behavior. As AI becomes more integrated into daily life and crucial decision making tasks, establishing trust in AI is essential. This necessitates a focus on enhancing transparency, explainability, fairness, and robustness within AI systems, which is also central to trustworthy AI.

While AI represents a new generation of automation technology with unique characteristics, research on trust in earlier forms of automation remains relevant. This historical perspective can inform the development of trust in AI by highlighting the important trust factors. Incorporating lessons from the past, the transition to AI additionally demands a renewed focus on ethical considerations, transparency, and user engagement to foster a deeper and more comprehensive trust.

#### 3.5.3 A framework of trust in automation

Early theoretical models of trust in automation focused on attributes related to automation’s performance or competence, such as reliability, robustness, capability, and predictability (Sheridan, 1988; Malle and Ullman, 2021). In these models, trust was primarily grounded in the systems’ technical performance and their ability to meet user expectations reliably. Trust varied directly with the system’s demonstrated competence in executing tasks (Malle and Ullman, 2021). However, the progress of AI has broadened the scope of research on trust determinants to include considerations of automation’s inferred intentions and the ethical implications of its actions (Malle and Ullman, 2021).

Individual differences are critical to human-automation trust. Some individuals apply frequently the *machine heuristic*, which is similar to trust propensity and represents the tendency to perceive automation as safer and more trustworthy than humans (Sundar and Kim, 2019). Moreover, an individual’s self-efficacy in using automation—the confidence in their ability to effectively utilize automation technologies—also plays a crucial role in shaping trust (Kraus et al., 2020); a higher sense of self-efficacy correlates with greater trust and willingness to use automated systems (Latikka et al., 2019). Furthermore, the degrees of familiarity and understanding of automated systems contribute to a more accurate evaluation of these systems’ competence, promoting a well-calibrated trust (Lee and See, 2004). Trust in automation, therefore, is a dynamic process, continuously recalibrated with the input of new information, knowledge, and experience (Muir, 1987).

Initial research on trust in automation adopted frameworks from interpersonal trust studies, positing a similar psychological structure in them (Muir, 1987). In practical research, factors affecting human-automation trust can also be categorized into trustor (human factors), trustee (automation factors), and the interaction context. This tripartite framework has been validated across various studies, affirming its applicability in understanding trust dynamics (Hancock et al., 2011; Hoff and Bashir, 2015; Drnec et al., 2016). Automation technology has evolved into the era of AI, inheriting characteristics of traditional automation while also exhibiting new features such as learning capabilities and adaptability. Factors influencing trust in AI based on this three-dimension framework are analyzed in detail in the next section from a sociocognitive perspective.

## 4 A three-dimension framework of trust in AI

AI, nested within the broad category of automation technology, benefits from existing trust research in automation, despite its unique characteristics. The algorithmic black-box nature of AI poses challenges in understanding its operational mechanisms, and its ability for autonomous learning compounds the difficulty in predicting its behavior. According to theories of technology acceptance, trust plays a pivotal role in the development and adoption of AI (Siau and Wang, 2018). Moreover, it is crucial for both enduring human collaborations and effective cooperation with AI (Xie et al., 2019). Furthermore, AI’s limitations in understanding human intentions, preferences, and values present additional challenges (Dafoe et al., 2021). Thus, research on trust in AI can guide the development of trustworthy AI, promote its acceptance and human interaction, and reduce the risks of misuse and disuse.

**Figure 1:**
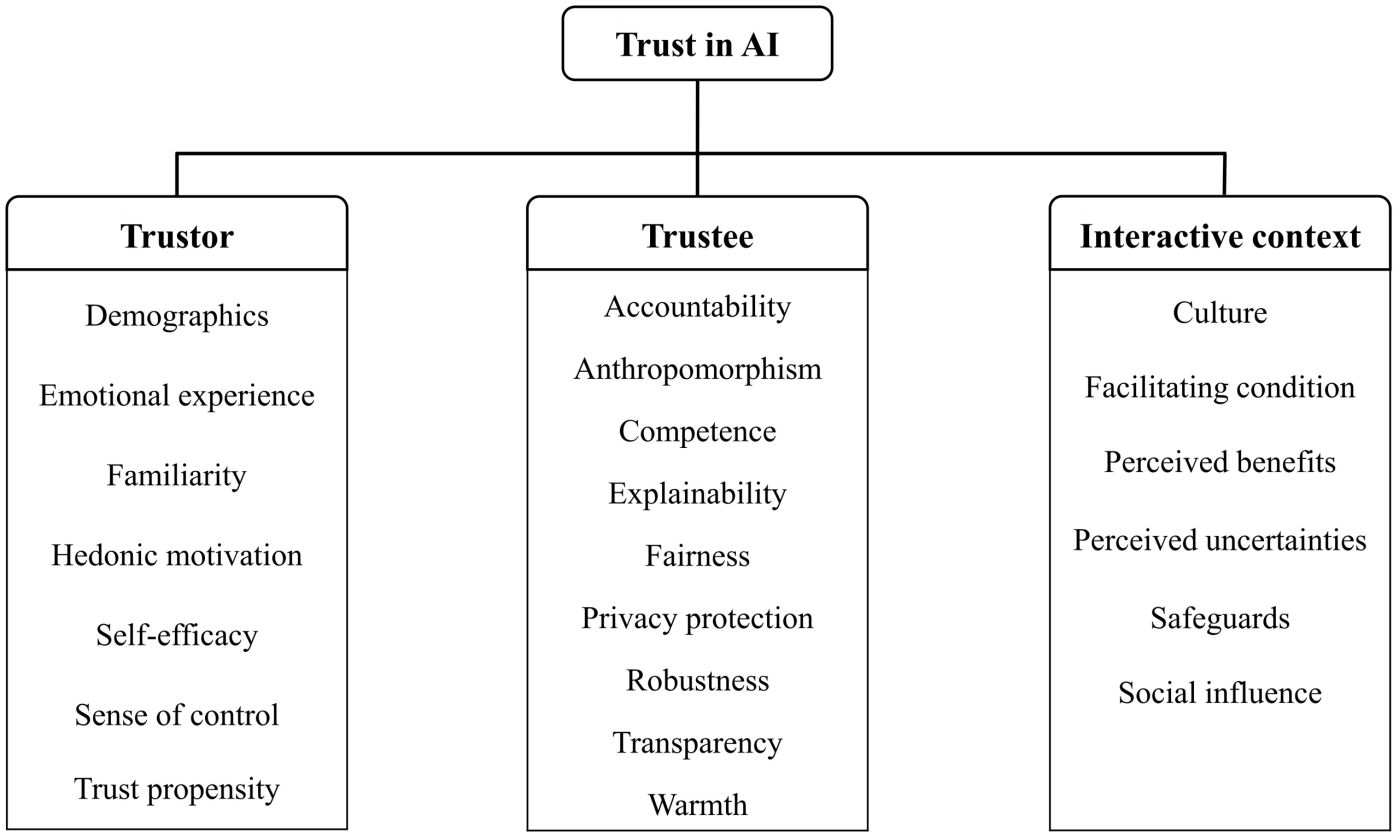
A three-dimension framework of trust that specifies the critical factors in each dimension that can affect trust in AI.

It is evident from our above review that trust, whether in interpersonal relationships or human-automation interactions, operates within a dyadic framework against an interactive context. Kaplan et al. (2021) also validated a similar framework through a meta-analysis of trust in AI, suggesting that the antecedents influencing trust in AI can be classified into three categories: human-related, AI-related, and context-related. In the following, we review factors influencing trust in AI related to these three dimensions, and Figure 1 shows what these factors are and to which dimension each of them belongs.

### 4.1 Factors related to the trustor

#### 4.1.1 Demographic variables

The impacts of demographic variables on trust in AI are complicated. In a recent worldwide survey study conducted in 17 countries, Gillespie et al. (2023) found that gender differences in trust toward AI were generally minimal, with notable exceptions in a few countries (i.e., the United States, Singapore, Israel, and South Korea). Regarding age, while a trend of greater trust among the younger generation was prevalent, this pattern was reversed in China and South Korea, where the older population demonstrated higher levels of trust in AI. Additionally, the data indicated that individuals possessing university-level education or occupying managerial roles tended to exhibit higher trust in AI, pointing to the significant roles of educational background and professional status in shaping trust dynamics. The study further showed pronounced cross-country variations, identifying a tendency for placing more trust in AI in economically developing nations, notably China and India. Previous research has also found that culture and social groups can influence trust in AI (Kaplan et al., 2021; Lee and Rich, 2021). Therefore, the impact of demographic variables on trust in AI may be profoundly influenced by socio-cultural factors.

#### 4.1.2 Familiarity and self-efficacy

An individual’s familiarity with AI, rooted in their knowledge and prior interactive experience, plays a pivotal role in trust formation (Gefen et al., 2003). Such familiarity not only reduces cognitive complexity by supplying essential background information and a cognitive framework, but also enables the formation of concrete expectations regarding the AI’s future behavior (Gefen, 2000). Additionally, a deeper understanding of AI can reduce the perceived risks and uncertainties associated with its use (Lu et al., 2019). Relatedly, AI use self-efficacy, or individuals’ confidence in their ability to effectively use AI, significantly influences acceptance and trust (REF). Familiarity and self-efficacy are both related to past interactive experience with AI, and both are positively correlated with a precise grasp of AI, thereby facilitating appropriate trust in AI.

#### 4.1.3 Hedonic motivation and emotional experience

Hedonic motivation, an intrinsic form of motivation, can lead people to use AI in pursuit of enjoyment. Venkatesh et al. (2012) recognized this by incorporating hedonic motivation into the expanded Unified Theory of Acceptance and Use of Technology (UTAUT) model as a key determinant of technology acceptance. This form of motivation is instrumental in increasing user satisfaction and enjoyment derived from AI, thereby positively influencing their attitudes toward technology and enhancing their intention to use it (Gursoy et al., 2019).

Emotional experience in the context of AI refers to the sense of security and comfort users feel when relying on AI, often described as emotional trust. It can reduce people’s perception of uncertainties and risks, and thus increase trust in AI. The acceptance and utilization of AI are guided by both cognitive judgments and affective responses. As such, it is crucial for trust research in AI to address both the cognitive and the emotional components (Gursoy et al., 2019). Specifically, emotional experience has been shown to directly impact the willingness to adopt AI-based recommendation systems, as seen in the context of travel planning (Shi et al., 2020).

#### 4.1.4 Sense of control

Sense of control represents individuals’ perception of their ability to monitor and influence AI decision-making processes. Dietvorst et al. (2018) found that algorithm aversion decreased when participants were allowed to adjust the outcomes of an imperfect algorithm, even if the adjustments were minimal. This finding underscores the importance of a sense of control in enhancing user satisfaction and trust in algorithms that are fundamental components of AI. Aoki (2021) found that AI-assisted nursing care plans that explicitly informed individuals that humans retained control over the decision-making processes significantly boosted trust in AI, compared to those who were not provided with this information. This highlights the importance of communicating human oversight in AI applications to enhance public trust. Similarly, Jutzi et al. (2020) found a favorable attitude toward AI in medical diagnosis when AI acted in a supportive capacity, reinforcing the value of positioning AI as an adjunct to human expertise.

#### 4.1.5 Trust propensity

The propensity to trust refers to stable internal psychological factors affecting an individual’s inclination to trust, applicable to both people and technology. Research indicates that individuals with high trust propensity are more inclined to place trust in others, including strangers, and hold a general belief in the beneficial potential of technology (Brown et al., 2004). This tendency enables them to rely on technological systems without extensive evidence of their reliability. Attitudes toward new technologies vary significantly; some individuals readily adopt new technologies, while others exhibit skepticism or caution initially. This variation extends to AI, where trust propensity influences acceptance levels (Chi et al., 2021). Furthermore, trust propensity may intersect with personality traits. For instance, individuals experiencing loneliness may show lower trust in AI, whereas those with a penchant for innovation are more likely to trust AI (Kaplan et al., 2021).

### 4.2 Factors related to the trustee

#### 4.2.1 Accountability

Because of the complexity and potential wide-ranging impacts of AI, accountability is a key factor in establishing public trust in AI. Fears that AI cannot be held responsible hinder trust in AI. Therefore, people need assurance that clear processes exist to handle AI issues and that specific parties, like developers, providers, or regulators, are accountable.

When people think that AI cannot be held accountable, they are less willing to let AI make decisions and tend to blame it less. Research has found that in the service industry when service providers make mistakes that result in customer losses, participants believe that the robot responsible for the mistake bears less responsibility compared to a human service provider, and the service-providing company should bear more responsibility (Leo and Huh, 2020). This occurs probably because people perceive that robots have poorer controllability over tasks. People are reluctant to allow AI to make moral decisions because AI is perceived to lack mind perception (Bigman and Gray, 2018). Bigman et al. (2023) found that algorithmic discrimination elicits less anger, with people showing less moral outrage toward algorithmic (as opposed to human) discrimination and being less inclined to blame the organization, but it does lower the evaluation of the company. This might be because people perceive algorithms as lacking prejudicial motivation.

#### 4.2.2 Anthropomorphism

Anthropomorphism, the tendency to ascribe human-like qualities to non-human entities such as computers and robots, significantly affects individuals’ trust in these technologies (Bartneck et al., 2009). Cominelli et al. (2021) found that robots perceived as highly human-like are more likely to be trusted by individuals. Beyond physical appearance and vocal cues, emotional expression is a crucial aspect of anthropomorphism. Troshani et al. (2021) found that robots exhibiting positive emotions are more likely to receive increased trust and investment from people. Similarly, Li and Sung (2021) demonstrated through a network questionnaire that anthropomorphized AI correlates with more positive attitudes toward the technology. Experimental studies have corroborated these findings, suggesting that psychological distance plays a mediating role in how anthropomorphism influences perceptions of AI (Li and Sung, 2021).

#### 4.2.3 Competence and warmth

The key to evaluating trustworthy AI is whether AI does what it claims to do (Schwartz et al., 2022). The claims of AI can be analyzed from two perspectives: one is whether it fulfills the functional requirements of its users, and the other is whether it demonstrates good intentions toward its users. This directly corresponds to the perceptions of competence and warmth of AI.

In both interpersonal trust and human-automation trust, competence and warmth are pivotal in shaping perceptions of trustworthiness (Kulms and Kopp, 2018). The stereotype content model (SCM) posits that stereotypes and interpersonal perceptions of a group are formed along two dimensions: warmth and competence (Fiske et al., 2002). Warmth reflects how one perceives the intentions (positive or negative) of others, while competence assesses the perceived ability of others to fulfill those intentions.

Trust and stereotype share a foundational link through the attitudes and beliefs individuals hold toward others (Kong, 2018). Moreover, in Mayer et al.’s (1995) trust model, the trustworthiness dimensions of ability and benevolence align closely with the competence and warmth dimensions in the SCM, respectively. Therefore, warmth and competence may be two core components of trustworthiness affecting interpersonal trust. The significance of these two dimensions is evident in their substantial influence on individuals’ evaluations and behaviors toward others (Mayer et al., 1995; Fiske, 2012).

Competence is the key factor influencing trust in automation (Drnec et al., 2016). When users observe errors in the automated system, their trust in it decreases, leading them to monitor the system more closely (Muir and Moray, 1996). However, an AI agent that is competent but not warm might not be trusted, because, in certain situations, intention is a crucial influencing factor of trust (Gilad et al., 2021). For instance, although users may recognize the technical proficiency of autonomous vehicles (AVs) in navigating complex environments, concerns that AVs may compromise safety for speed or prioritize self-preservation in emergencies can undermine trust (Xie et al., 2019). Thus, AI needs to demonstrate good intentions to build trust. This is exemplified by AI’s social intelligence, such as understanding and responding to user emotions, which significantly bolsters trust in conversational agents (Rheu et al., 2021). Moreover, trust in AI is generally lower in domains traditionally dominated by human expertise, potentially due to concerns about the intentions of AI (Lee, 2018).

#### 4.2.4 Privacy protection

The rapid development of AI, facilitated greatly by network technology, raises privacy concerns, especially when third parties access data through networks without user consent, risking privacy misuse (Featherman and Pavlou, 2003). Additionally, while AI’s ability to tailor services to individual needs can enhance user satisfaction, this often requires accessing personal information, thus creating a dilemma between personalization benefits and privacy risks (Guo et al., 2016). Network technologies have amplified privacy risks, resulting in individuals losing control over the flow of their private data. As a result, privacy concerns play a crucial role in establishing online trust, and internet users are highly concerned about websites’ privacy policies, actively seeking clues to ensure the protection of their personal information (Ang et al., 2001). Research has found that providing adequate privacy protection measures directly influences people’s trust in AI and their willingness to use it (Vimalkumar et al., 2021; Liu and Tao, 2022).

#### 4.2.5 Robustness and fairness

Sheridan (1988) argued that robustness should be an important determinant of trust. The robustness of AI refers to the reliability and consistency of its operations and results, including its performance under diverse and unexpected conditions (High-Level Expert Group on Artificial Intelligence (AI HLEG), 2019). The fairness of AI involves treating all users equitably, making unbiased decisions, and not discriminating against any group (Shin and Park, 2019). Robustness is a key factor influencing trust in AI. Rempel et al. (1985) identified three components of trust from a dynamic perspective, including predictability (the consistency of actions over time), dependability (reliability based on past experience), and faith (belief in future behavior). Based on the definitions, these components also correspond to the formation of the perception of robustness. Compared to trust in humans, building trust in AI takes more time; moreover, when AI encounters problems, the loss of trust in it happens more rapidly (Dzindolet et al., 2003). Furthermore, the simpler the task in which the error occurs, the greater the loss of trust (Madhavan et al., 2006).

Because robustness and fairness are vulnerable to data bias, from both theoretical and practical standpoints, these two factors are closely related. Robustness serves as a crucial foundation for fairness, with the presence of discrimination and bias often signaling a lack of robustness. For example, the training data used for developing large language models often contain biases, and research has found that ChatGPT replicates gender biases in reference letters written for hypothetical employees (Wan et al., 2023). Such disparities underscore the importance of aligning AI with human values, as perceived fairness significantly influences users’ trust in AI technologies (Angerschmid et al., 2022).

#### 4.2.6 Transparency and explainability

Users need to understand how and why AI makes specific decisions, which corresponds, respectively, to transparency and explainability, before trusting in it. However, this is not an easy task for AI practitioners and stakeholders. Because of the mechanisms of algorithms, particularly the opacity of neural networks, it is difficult for humans to fully comprehend their decision-making process (Shin and Park, 2019).

Transparent AI models with clear explanations of their decision-making processes help users gain confidence in the system’s capabilities and accuracy. Moreover, transparency in AI design and implementation helps identify potential sources of bias, allowing developers to address these issues and ensure the AI system treats all users fairly. The concepts of transparency and explainability are deeply interconnected; explainability, in particular, plays a crucial role in reducing users’ perceived risks associated with AI systems (Qin et al., 2020). Additionally, providing reasonable explanations after AI errors can restore people’s trust in AI (Angerschmid et al., 2022).

That said, the impact of transparency and explainability on trust in AI shows mixed results. Leichtmann et al. (2022) found that displaying AI’s decision-making process through graphical and textual information enhances users’ trust in the AI program. However, Wright et al. (2020) found no significant difference in trust levels attributed to varying degrees of transparency in simulated military tasks for target detection. Furthermore, in a task of using AI assistance to rate movies, Schmidt et al. (2020) observed that increased transparency in AI-assisted movie rating tasks paradoxically reduced user trust.

Therefore, the relationship between transparency and trust in AI is intricate. Appropriate levels of transparency and explainability can enhance people’s trust in AI, but excessive information might be confusing (Kizilcec, 2016), thereby reducing their trust in AI. The absence of clear operational definitions for AI’s transparency and explainability complicates the determination of the optimal transparency levels that effectively build trustworthy AI. In general, lack of transparency indeed hurts trust in AI, but high levels of transparency do not necessarily lead to good results.

### 4.3 Factors related to the interactive context of trust

#### 4.3.1 Perceived uncertainties and benefits

AI is surrounded by various unknowns, including ethical and legal uncertainties, that are critical evaluations of the interactive environment. Lockey et al. (2020) emphasized uncertainties as a key factor influencing trust in AI. Similarly, Jing et al. (2020) conducted a literature review and discovered a negative correlation between perceived uncertainties and risks with the acceptance of autonomous driving. Furthermore, perceived uncertainties in the application of AI vary across different applications, particularly pronounced in medical expert systems and vehicles (Yang and Wibowo, 2022).

Perceived benefits, such as time savings, cost reductions, and increased convenience, highlight the recognized advantages of using AI (Kim et al., 2008). Liu and Tao (2022) found that perceived benefits, such as usefulness, could facilitate the use of smart healthcare services. Although perceived benefits can be viewed as characteristics of AI the trustee, they can also be highly socially dependent, mainly because the impacts of these benefits are not uniform across all users: For instance, while AI applications may enhance work efficiency for some, they could pose a risk of unemployment for others (Pereira et al., 2023). Therefore, perceived benefits are intricately linked to the social division of labor, underscoring their importance within the broader interactive context of AI usage.

#### 4.3.2 Safeguards

Drawing from the concept of institution-based trust, safeguards are understood as the belief in existing institutional conditions that promote responsible and ethical AI usage (McKnight et al., 1998). Because AI is perceived as lacking agency and cannot be held accountable for its actions (Bigman and Gray, 2018), safeguards play a crucial role in ensuring human trust in AI.

Lockey et al. (2020), however, found that a mere 19–21% of Australians considered the current safety measures adequate for AI’s safe application, underscoring a significant trust gap. Their analysis further showed that perception of these safeguards was a strong predictor of trust in AI. In today’s AI landscape, establishing legal frameworks to protect human rights and interests is crucial for fostering trust. A prime example is the European Union’s General Data Protection Regulation (GDPR). Enacted in 2018, GDPR introduces stringent privacy protections and sets clear standards for algorithmic transparency and accountability (Felzmann et al., 2019).

#### 4.3.3 Social influence and facilitating condition

Social influence is defined by the extent to which an individual perceives endorsement of specific behaviors by their social network, including encouragement from influential members to adopt new technologies (Gursoy et al., 2019). It is a crucial construct in the UTAUT (Venkatesh et al., 2003). Social influence theory posits that individuals tend to conform to the norms and beliefs of their social network (Shi et al., 2020). When individuals perceive that the use of AI is socially acceptable, they are more likely to experience positive emotions toward it, leading to an increase in their emotional trust in AI.

Facilitating condition is another critical variable in the UTAUT model, referring to the extent to which individuals perceive organizational, group, or infrastructural support in using AI (Venkatesh et al., 2003). Chi et al. (2021) found that facilitating robot-use conditions could improve users’ trust in social robots in service scenarios.

#### 4.3.4 Culture

Cultural factors can significantly influence trust and acceptance of AI. For instance, cultures with high uncertainty avoidance are more inclined to trust and depend on AI (Kaplan et al., 2021), and the level of trust in AI also varies between individualistic and collectivistic cultures (Chi et al., 2023). Moreover, cultural influences may interact with economic factors to affect AI trust. Gillespie et al. (2023) found that individuals in the emerging economies, such as Brazil, India, China, and South Africa, exhibit higher levels of trust, in comparison to developed nations, such as the United Kingdom, Australia, Japan, and France. Furthermore, the impact of culture on AI trust can be mediated through social influence, highlighting the importance of social norms (Chi et al., 2023).

## 5 Implications for enhancing trustworthy AI

While intention is pivotal in interpersonal trust, competence is paramount in human-automation trust. Nonetheless, research on trust in AI encompasses both competence and intention, indicating that AI is perceived through a combination of human-like and automated characteristics, reflecting a sociocognitive perspective on trust in AI (Kulms and Kopp, 2018). Understanding how trust in AI forms from this perspective and integrating the resulting knowledge into the design and applications of AI systems will be critical to foster their effective use and the collaboration between humans and AI.

The proposed three-dimension framework of trust in AI not only encompasses the desired characteristics of AI but also emphasizes enhancing AI literacy among users and refining the interactive context. The framework highlights users’ expectations of AI and can help developers and managers grasp user concerns and needs. In addition, given the imperative to reduce perceived uncertainties associated with AI, it becomes critical to address concerns related to privacy protection in AI applications, ensure accountability, and meet the demand for enhanced safeguard measures.

The three-dimension framework also provides a solid foundation for developing ethical standards and policies that can enhance trustworthy AI. In social psychology, competence and warmth are critical for assessing trustworthiness. These dimensions are equally vital in evaluating AI. Specifically, robustness and safety illustrate competence, whereas fairness and privacy protection embody warmth. Thus, in formulating ethical standards for trustworthy AI, we would recommend focusing on the key dimensions of competence and warmth. For example, in developing and deploying AI applications, it is critical to conduct an ethical evaluation based on their competence and warmth. This evaluation ensures that the applications are functionally effective and possess benevolent intentions toward humanity. In addition, as AI technology advances, its potential to infringe upon human rights intensifies, underscoring the increasing importance of evaluating its warmth.

Recognizing how individual characteristics influence trust in AI can guide the development of personalized and adaptive AI interaction strategies. These strategies, tailored to meet the specific needs and preferences of diverse users, can foster a sustained and appropriate trust in AI. While some individuals may place excessive trust in AI because of a high trust propensity, others, hindered by limited understanding of AI and a lower sense of self-efficacy, may demonstrate a lack of trust. Lockey et al. (2020) discovered a widespread desire among individuals to learn more about AI. Therefore, in developing trustworthy AI, it is crucial to acknowledge the varying levels of trust people have toward AI and to devise effective communication strategies, engaging in AI education to bridge this knowledge gap.

Moreover, hedonic motivation plays a critical role in shaping trust in AI, with the potential to cause users to overtrust AI systems. For example, algorithms behind short video apps often leverage this motivation, leading to excessive requests for user data (Trifiro, 2023). Despite users’ general inclination to protect their privacy, they often adopt a pragmatic approach toward privacy protection, a discrepancy referred to as the *privacy paradox* (Sundar and Kim, 2019). Therefore, it is essential to be vigilant against the overtrust in AI that may result from an excessive pursuit of enjoyment and entertainment.

Furthermore, power asymmetry often results in trust asymmetry. The prevailing trust in AI serves as a pertinent example of this asymmetry, where interactions with AI-driven technologies may engender a perceived sense of power or dominance among users. Such perceptions significantly influence the dynamics of trust in AI (Fast and Schroeder, 2020). Consequently, the influence of this sense of power on human interactions with AI necessitates further investigation.

Gaining insight into the factors affecting AI’s trustworthiness enables a more sophisticated approach to identifying and managing the inherent risks associated with its application. Notably, anthropomorphism, the attribution of human-like qualities to AI, significantly influences users’ emotional trust, potentially enhancing AI’s acceptance and trustworthiness (Glikson and Woolley, 2020). Anthropomorphized AI might be more readily accepted and trusted by users, yet this could also mask its inherent limitations and potential risks. Further, attributing human traits to AI can lead to unrealistic expectations about its capabilities, including agency and moral judgment, thereby fostering misconceptions about its competence. Thus, cultivating trustworthy AI requires ensuring that users possess an accurate understanding of AI’s anthropomorphic features.

Policymakers focused on trustworthy AI must recognize the significant influence of social, organizational, and institutional factors in shaping AI perceptions within the interactive context of trust. The mass media plays a pivotal role in influencing public attitudes toward AI, either by highlighting uncertainties or by raising awareness of new technological advancements. A series of studies have shown the significant role of media in promoting emerging technologies (Du et al., 2021). Media headlines can influence people’s emotional responses, thereby affecting their willingness to adopt technology (Anania et al., 2018). Mass media can also influence trust in AI by impacting social influence and self-efficacy. Given these dynamics, regulating mass media to ensure accurate representation of AI is crucial. Policymakers should additionally prioritize the establishment of clear laws and regulations, define responsibilities for AI failures, and engage in transparent communication with the public to mitigate perceived uncertainties.

For example, trust in autonomous vehicles is dynamic (Luo et al., 2020) and easily swayed by mass media (Lee et al., 2022). Furthermore, media portrayals often lack objectivity, with companies overstating autonomy levels in promotions, whereas media primarily reports on accidents. Therefore, ensuring balanced and factual media representations is essential to foster an environment where people can develop informed trust in autonomous vehicles. Moreover, implementing sensible legislation and regulations, as well as clarifying responsibility in accidents involving autonomous vehicles, is vital for public endorsement.

High-Level Expert Group on Artificial Intelligence (AI HLEG) (2019) delineated seven crucial requirements for trustworthy AI: human agency and oversight, technical robustness and safety, privacy and data governance, transparency, diversity, non-discrimination and fairness, societal and environmental wellbeing, and accountability. The factors influencing trust in AI as we have reviewed (see Figure 1) are generally consistent with these requirements. High-Level Expert Group on Artificial Intelligence (AI HLEG) (2019) also proposes communication, education, and training as key non-technical strategies for promoting trustworthy AI, again consistent with our recommendations derived from the literature.

Generative AI has emerged as the most noteworthy development in AI technologies in recent years, with products such as GPT and Sora showing impressive capabilities in content generation and analysis (Yang et al., 2024). For instance, videos created by Sora can be indistinguishably realistic. Even large language models are being utilized to explain other AI models, enhancing AI’s explainability (Bills et al., 2023). As AI’s capabilities grow, so does its impact on society, including potential negative effects, such as the ease of generating fraudulent content through generative AI. Concurrently, governments worldwide are introducing laws and regulations to guide AI development responsibly. On March 13, 2024, the European Union passed *The AI Act*, the world’s first comprehensive regulatory framework for AI (European Parliament, 2024). It categorizes AI usage by risk levels, banning its use in certain areas such as social scoring systems and the remote collection of biometric information and highlighting the importance of fairness and privacy protection. While the competence of AI is advancing, skepticism about its warmth also grows. Simultaneously, the emphasis on its warmth and the need for safeguards will increase.

Overall, we review factors influencing trust formation from the user’s perspective via a three-dimension model of trust in AI. The framework, with its detailed examination of factors impacting the trustor, the trustee, and their interaction context, is instrumental in guiding the creation of targeted educational and training programs that are essential for enabling users to understand and engage with AI more effectively. Furthermore, trustworthy AI could benefit from the adoption of trust measurement methods to assess the effectiveness of these initiatives. These assessments should include both subjective self-report methods and objective indicators of engagement with AI technologies, including reliance, compliance, and decision-making behavior and time.

## 6 Summary and conclusion

This article provides a comprehensive review and analysis of factors influencing trust in AI and offers insights and suggestions on the development of trustworthy AI. The three-dimension framework of trust is applicable for understanding trust in interpersonal relationships, human-automation interactions, and human-AI systems. The framework can also help understand user needs and concerns, guide the refinement of AI system designs, and aid in the making of policies and guidelines on trustworthy AI. All of these shall lead to AI systems that are more trustworthy, increasing the likelihood for people to accept, adopt, and use them properly.

## Author contributions

YL: Writing – original draft. BW: Writing – original draft. YH: Writing – original draft. SL: Writing – review & editing.
